# Reduced autobiographical memory specificity is associated with impaired discrimination learning in anxiety disorder patients

**DOI:** 10.3389/fpsyg.2015.00889

**Published:** 2015-07-01

**Authors:** Bert Lenaert, Yannick Boddez, Bram Vervliet, Koen Schruers, Dirk Hermans

**Affiliations:** ^1^Faculty of Psychology and Educational Sciences, Centre for the Psychology of Learning and Experimental Psychopathology, University of LeuvenLeuven, Belgium; ^2^Faculty of Health, Medicine and Life Sciences, School for Mental Health and Neuroscience, Maastricht UniversityMaastricht, Netherlands

**Keywords:** associative learning, discrimination learning, generalization, autobiographical memory, anxiety

## Abstract

Associative learning plays an important role in the development of anxiety disorders, but a thorough understanding of the variables that impact such learning is still lacking. We investigated whether individual differences in autobiographical memory specificity are related to discrimination learning and generalization. In an associative learning task, participants learned the association between two pictures of female faces and a non-aversive outcome. Subsequently, six morphed pictures functioning as generalization stimuli (GSs) were introduced. In a sample of healthy participants (Study 1), we did not find evidence for differences in discrimination learning as a function of memory specificity. In a sample of anxiety disorder patients (Study 2), individuals who were characterized by low memory specificity showed deficient discrimination learning relative to high specific individuals. In contrast to previous findings, results revealed no effect of memory specificity on generalization. These results indicate that impaired discrimination learning, previously shown in patients suffering from an anxiety disorder, may be—in part—due to limited memory specificity. Together, these studies emphasize the importance of incorporating cognitive variables in associative learning theories and their implications for the development of anxiety disorders. In addition, re-analyses of the data (Study 3) showed that patients suffering from panic disorder showed higher outcome expectancies in the presence of the stimulus that was never followed by an outcome during discrimination training, relative to patients suffering from other anxiety disorders and healthy participants. Because we used a neutral, non-aversive outcome (i.e., drawing of a lightning bolt), these data suggest that learning abnormalities in panic disorder may not be restricted to fear learning, but rather reflect a more general associative learning deficit that also manifests in fear irrelevant contexts.

## Introduction

The role of associative learning in the development and the course of anxiety disorders is well-established (Mineka and Zinbarg, 2006). For example, during a robbery, a number of neutral stimuli (e.g., the color of the sweater the bank robber was wearing) may become associated with an aversive outcome (e.g., suffering violent force). As a result, these previously neutral stimuli may come to trigger fear.

However, only a small subset of individuals who are confronted with an aversive learning experience will develop anxiety complaints (e.g., Breslau, 2009). Individual differences in genetic predisposition (Martin et al., 2009), psychological constitution, and learning history (e.g., Zvolensky et al., 2005) jointly determine whether or not learning episodes will result in psychopathology. With respect to learning history, it has been shown that individual differences in, for example, extinction learning predict subsequent onset of anxiety symptomatology. Lommen and colleagues tested 249 Dutch soldiers before their deployment to Afghanistan (Lommen et al., 2013). During the extinction phase, a neutral stimulus (S+) that was previously paired with an aversive outcome was presented in the absence of the outcome, typically resulting in a reduction in responding to the S+. Interestingly, results showed that post-deployment anxiety symptoms were more severe in individuals who displayed impaired extinction learning before deployment (see also Guthrie and Bryant, 2006).

In addition to reduced extinction learning, other learning abnormalities have been implicated in the development of pathological anxiety. For instance, impaired discrimination learning between a stimulus (S+) that predicts a certain outcome and a stimulus (S−) that predicts the absence of the outcome has been found in individuals suffering from an anxiety disorder (e.g., Grillon and Morgan, 1999; Lissek et al., 2009), as well as in individuals with subclinical levels of anxiety (e.g., Chan and Lovibond, 1996; Haddad et al., 2012; Arnaudova et al., 2013; Gazendam et al., 2013). Further, overgeneralization of learned responding has been demonstrated in panic disorder, generalized anxiety disorder, and post-traumatic stress disorder (Lissek et al., 2010, 2014; Lissek and Grillon, 2012). Generalization refers to the observation that learning about one stimulus (e.g., S+) typically results in a tendency to respond to stimuli that are perceptually similar to the S+ (generalization stimuli or GSs). Overgeneralization to these GSs has a large share in the debilitating impact of anxiety disorders on daily life. In panic disorder with agoraphobia, for instance, daily functioning becomes increasingly hampered when an individual starts avoiding all or most public places, and not just the place where he or she first experienced a panic attack.

The documentation of differences in associative learning between healthy individuals and individuals suffering from clinical or subclinical anxiety represents an important step toward a better understanding of anxiety and its development, but more steps need to be taken. First, it remains largely unclear whether these learning deficits are antecedents or consequences of pathological anxiety (vulnerability factors or diagnostic marker, respectively; Beckers et al., 2013), so more longitudinal work is critically needed (e.g., Lommen et al., 2013; Lenaert et al., 2014). Second, knowledge about the mechanisms underlying these group differences in associative learning is crucial in order to inform theories about the origin of anxiety disorders, and to offer new leads for treatment and targeted prevention.

In the present study, we focus on this second aspect and investigate the effect of memory on discrimination learning and generalization in a sample of healthy participants (Study 1), as well as in individuals suffering from an anxiety disorder (Study 2). Memory plays an important role in associative learning, in that various aspects of remembering and forgetting determine the amount of learned information that is expressed in behavior (e.g., Bouton and Moody, 2004). For instance, Riccio and colleagues showed that generalization tends to increase as a function of the time interval between learning and testing (Riccio et al., 1994; Jasnow et al., 2012). This increase in generalization over time is considered to be a memory phenomenon. In this view, a memory of a certain stimulus is conceptualized as being composed of multiple attributes (e.g., shape, texture, color, etc.). As time passes, some of these attributes may be more difficult to retrieve from memory, which may lead to decreased discriminability of the original stimulus from novel, more, or less resembling stimuli, resulting in increased generalization. Generalization thus depends on the retrieval of memorized experiences.

Autobiographical memory research has demonstrated that people vary in the ability to retrieve detailed features of memorized experiences. For instance, it has been convincingly shown that certain clinical groups experience difficulties in retrieving specific memories and tend to retrieve memories that cover whole categories of events (e.g., post-traumatic stress disorder, clinical depression; Williams et al., 2007). Several mechanisms are assumed to be involved in reduced memory specificity. For instance, according to the functional avoidance hypothesis, the emotional impact of painful memories of life events may be diminished by reducing the specificity of these memories. Although this strategy may be adaptive over the short-term in that it prevents confrontation with specific details of negative or painful memories, reduced memory specificity has been associated with long-term negative outcomes (e.g., a more negative course of depression; Sumner et al., 2010). It is noteworthy that, for most anxiety disorders, no consistent evidence exists for reduced memory specificity (Williams et al., 2007). However, our main objective was not to investigate these between-group differences. Rather, we aimed to examine whether autobiographical memory specificity is related to individual differences in discrimination learning and generalization within a sample of healthy individuals on the one hand, and a sample of anxiety disorder patients on the other hand. Interestingly, in a previous investigation, we found that first-year psychology students who were characterized by limited memory specificity showed higher levels of generalization of learning to perceptually similar GSs, relative to their high specific counterparts (Lenaert et al., 2012).

Building on this proof of principle study, we investigated the relationship between memory specificity on the one hand, and generalization and discrimination learning on the other hand in Study 1 and Study 2. We tested individual differences in memory specificity using the Autobiographical Memory Test (AMT; Williams and Broadbent, 1986). In this cue-word paradigm, participants are instructed to come up with a specific memory of a personally experienced event in response to a number of cue words (e.g., proud, disappointed). A specific memory is defined as a memory that refers to an event that only took place once and lasted no longer than 24 h (e.g., “I was proud of my daughter when she got her driving license 3 months ago”). Discrimination learning and generalization were assessed using an associative learning task consisting of two phases. In the discrimination learning phase, a neutral stimulus (S+) was contingently paired with a non-aversive outcome, whereas a second neutral stimulus (S−) was never paired with the outcome. Discriminatory learning is evident if the S+ elicits higher expectancies of the outcome than the S−. The S+ and the S− were pictures of neutral female faces. In the subsequent generalization test phase, we tested the extent of generalization of learned responses to a number of pictures of female faces that differed in their perceptual similarity with the original S+/S−.

Finally, we were also interested in differences in associative learning between healthy individuals and individuals suffering from an anxiety disorder. In Study 3, a re-analysis of the data collected in Study 1 and 2 was performed to investigate whether learning abnormalities that have been previously shown in patients suffering from an anxiety disorder also manifest in a neutral associative learning task with a fear irrelevant outcome.

## Study 1

In study 1, we tested the relationship between memory specificity and discrimination learning and generalization in a sample of healthy participants. Following the reasoning explained above, accurate discrimination between similar past experiences, such as S+/S− presentations, requires the encoding and/or retrieval of event-specific knowledge. Hence, we predicted that individuals who are characterized by limited memory specificity would evidence deficient discrimination learning (i.e., slower learning of the S+/S− differentiation on a trial by trial basis, and weaker differentiation by the end of acquisition) relative to their high specific counterparts. Further, we hypothesized that memory specificity would be associated with the extent of generalization to perceptually similar stimuli. More precisely, we predicted that high specific participants would show lower levels of generalization relative to low specific participants.

### Method

#### Participants

Twenty-nine healthy participants (22 women) entered this study, who—on the basis of self-report—had to be free of any current or past anxiety or depressive disorder. Their mean age was 40.9 (*SD* = 16.9, age range: 17–86). All participants were Caucasian and had the Dutch nationality. The study was approved by the Medical Ethics Committee, Maastricht University, The Netherlands. All participants gave informed consent.

#### Apparatus and stimuli

**Figure 1:**
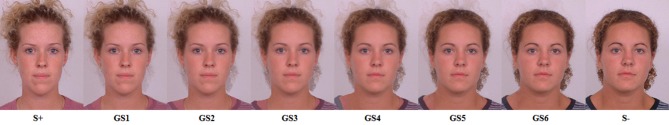
**The S+, the S−, and the 6 GSs**. For half of participants, the S+, the S−, and the corresponding GSs were reversed.

Stimuli were presented on a black background in the center of a laptop computer screen (38.1 cm). The associative learning task was programmed with Affect 4.0 software (Spruyt et al., 2010). The S+ and S− were pictures of neutral human female faces (DeBruine, 2005). For half of the participants, one picture of a female face served as S+, whereas the other picture served as S−. For the other participants, this was reversed. The pictures were presented 95 mm wide and 127 mm high (360 × 480 pixels). The GS were obtained by transforming the S+ into the S− in six gradual steps using specialized software, resulting in six GS that resembled the S+ (S−) to a greater or lesser extent (see Figure 1). A drawing of a white lightning bolt, which measured 45 by 28 mm, served as the outcome. The experimental trials consisted of the presentation of a picture of a female face (S+, S− or GS), which was immediately followed by the outcome only after S+ presentations. The lightning bolt was presented for 1500 ms. The inter trial interval was set to 3000 ms.

#### Measures

#### Outcome expectancy

During all S+, S−, and GS trials, participants were requested to indicate their expectancy of the outcome on an 11-point scale ranging from 0 to 10 (with 0 meaning “I am absolutely sure no lightning bolt will follow”, and 10 meaning “I am absolutely sure a lightning bolt will follow”). The scale was presented at the bottom of the computer screen. Participants indicated their expectancy by moving a red dot across the scale using the left and right arrows, and confirmed their chosen expectancy rating by hitting “Enter.” There was no time limit for this response.

#### Autobiographical memory test

Memory specificity was assessed by a written version of the AMT (Williams and Broadbent, 1986), consisting of five positive cue-words and five negative cue-words presented in strictly alternating order. The cue-words were: pleasant, angry, interested, hurt, proud, maddened, sociable, clumsy, enthusiastic, and disappointed^1^. Participants were given a booklet with written instructions, which were repeated verbally by the experimenter. They were asked to generate a specific memory in response to each of the cue-words. A specific memory was defined as a memory of a personally experienced event that occurred only once, within the course of 1 day. Examples of specific (e.g., “I was happy with the beautiful wristwatch I got for my birthday 3 weeks ago”), and non-specific memories (e.g., “I am always happy when I get gifts for my birthday”) were provided. A time limit of 60 s was set for each cue-word, after which participants were requested to immediately stop writing. Afterwards, the memories were coded as either specific or non-specific. The latter code was assigned to memories for events that occurred more than once (categorical memory), lasted longer than a day (extended memory), or to responses that did not represent an actual memory (e.g., reflections about the present or the future). The memory specificity score for each individual was calculated as the proportion of specific memories relative to the total number of responses given.

#### Procedure

Both verbal and written instructions were given prior to the experiment. Participants were informed that pictures of female faces would appear on the computer screen, and that these would sometimes be followed by a pictogram of a lightning bolt. They were instructed that the goal of the task was to figure out which picture would be followed by a lightning bolt.

The associative learning task consisted of a pre-acquisition phase, an acquisition phase, and a generalization phase. During pre-acquisition, both the S+ and the S− were presented three times, each without being followed by the outcome. During the acquisition or *discrimination learning* phase, the S+ and the S− were presented 12 times each, with the S+ being followed by the outcome nine times (75% reinforcement). During the *generalization* phase, three consecutive blocks of ten trials were presented. Each block consisted of two S+ trials, two S− trials, and one trial for each of the six GSs. The outcome was presented once in each block, after one of the two S+ trials. This was done to prevent rapid extinction during the test of generalization. Throughout the experiment, trials were presented in semi-random order, with the restriction of no more than two consecutive trials of the same type. After the learning task, all participants completed the AMT.

#### Data analysis

The data (see Supplementary Material) from the associative learning task were analyzed using repeated measures multivariate analysis of variance (MANOVA). Outcome expectancy served as the dependent variable. In order to test the relationship between memory specificity on the one hand, and discrimination learning and generalization on the other hand, a median split procedure was used to differentiate participants high and low in autobiographical memory specificity. Predictors were Specificity (low specific, high specific) as between-subjects factor, and Stimulus (S+, S−) and Trial (1–12) as within-subjects factors. Main analyses of interest looked at Specificity × Stimulus, and Specificity × Stimulus × Trial interactions. Comparative analyses were performed to investigate differences in outcome expectancy as a function of memory specificity for the S+/S− differentiation, as well as for the S+ and S− separately. In addition to this approach, we also calculated an index of discrimination learning as the difference in outcome expectancy between the S+ and the S− over all trials, with larger differences pointing to better discrimination learning. This allowed us to investigate the relationship between memory specificity and discrimination learning in a continuous fashion. With respect to generalization, predictors were Specificity (low specific, high specific) as between-subjects factor, and Block (1–3) Stimulus (S+, GS1, GS2, GS3, GS4, GS5, GS6, S−) as within-subjects variables. Main analyses of interest looked at a Specificity × Stimulus interaction. All main analyses were also performed with probit transformed data to produce more normal data distributions. Results remained unchanged and are not reported. Finally, for all three studies, power estimates were calculated for medium effect sizes and with correlations among repeated measures ranging between 0.4 and 0.5, which was typical for our data (Faul et al., 2007). For study 1, based on a sample of 29, power estimates were on the low side (ranging between 0.44 and 0.52). For Study 2, based on a sample of 49, power estimates ranged between 0.67 and 0.76. For study 3, where three groups were compared, power estimates ranged between 0.77 and 0.85.

### Results

#### Memory specificity

The mean proportion of specific memories was 0.68 (*SD* = 0.31, range: 0–1). A median split procedure was used to differentiate participants high and low in autobiographical memory specificity. The mean proportion of specific memories was 0.46 (*SD* = 0.29, range: 0–0.75) for the low specific individuals (*N* = 15), and 0.92 (*SD* = 0.08, range: 0.78–1) for the high specific individuals (*N* = 14).

#### Memory specificity and discrimination learning

**Figure 2:**
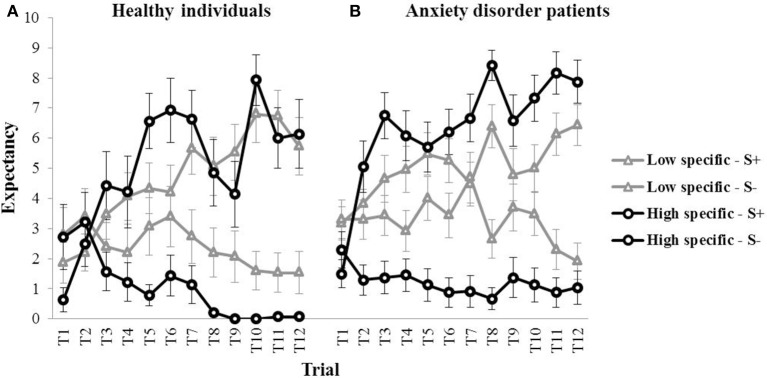
**Mean outcome expectancy ratings for S+ and S− (+SEM) throughout the discrimination learning phase (trial 1–trial 12) as a function of memory specificity (high vs. low memory specificity), for (A) healthy individuals (left panel, Study 1), and (B) anxiety disorder patients (right panel, Study 2)**.

Figure 2 (left panel) provides a graphical representation of the outcome expectancies for all S+ and S− trials during discrimination training for both the high specific and the low specific healthy participants (means for all trials are presented in Table 1). The figure suggests differences in learning between groups, with *slower* acquisition of the S+/S− discrimination on a trial-by-trial basis, as well as a *weaker* S+/S− discrimination by the end of acquisition in the low specific group relative to the high specific group. A repeated measures MANOVA with Specificity (low specific, high specific) as between-subjects variable, and Stimulus (S+, S−) and Trial (1–12) as within-subjects variables revealed a main effect of Stimulus, *F*_(1, 27)_ = 41.91, *p* < 0.001, partial η^2^ = 0.61, and a Stimulus × Trial interaction, *F*_(11,17)_ = 4.20, *p* = 0.004, partial η^2^ = 0.29. However, the Specificity × Stimulus interaction was only supported at trend level, *F*_(1, 27)_ = 3.52, *p* = 0.071, partial η^2^ = 0.11. We also found no Specificity × Stimulus × Trial interaction, *F*_(11,17)_ = 0.89, *p* = 0.564, partial η^2^ = 0.05. However, these analyses may have lacked power. Finally, the correlation between the index of discrimination learning and autobiographical memory specificity was in the expected direction, but was not significant, *r*_(28)_ = 0.25, *p* = 0.185. Again, this may be attributable to a relatively small sample size, as power was indeed quite low for this analysis (0.26). In sum, although visual inspection of Figure 2 (left panel) suggests slower and weaker discrimination learning in low specific individuals relative to high specific individuals, statistical analyses provided no evidence for differences in discrimination learning as a function of memory specificity.

**Table 1:** **Mean outcome expectancies for the S+ and the S− throughout acquisition (trial 1–trial 12) for the healthy group (Study 1), and the anxiety disorder group (Study 2) as a function of memory specificity (low specific vs. high specific)**.

**Trial**	**T1**	**T2**	**T3**	**T4**	**T5**	**T6**	**T7**	**T8**	**T9**	**T10**	**T11**	**T12**
**STUDY 1: HEALTHY GROUP**												
Low specific S+	1.87	2.20	3.47	4.07	4.33	4.20	5.67	5.07	5.53	6.80	6.73	5.73
Low specific S−	2.80	3.40	2.40	2.20	3.07	3.40	2.73	2.20	2.07	1.60	1.53	1.53
High specific S+	0.64	2.50	4.43	4.21	6.57	6.93	6.64	4.86	4.14	7.93	6.00	6.14
High specific S−	2.71	3.21	1.57	1.21	0.79	1.43	1.14	0.21	0.00	0.00	0.07	0.07
**STUDY 2: ANXIETY DISORDER GROUP**												
Low specific S+	3.17	3.83	4.65	4.96	5.48	5.26	4.48	6.39	4.78	5.00	6.13	6.43
Low specific S−	3.30	3.30	3.43	2.91	4.00	3.43	4.70	2.65	3.70	3.48	2.30	1.91
High specific S+	1.50	5.04	6.75	6.08	5.71	6.21	6.67	8.42	6.58	7.33	8.17	7.88
High specific S−	2.29	1.29	1.38	1.46	1.13	0.88	0.92	0.67	1.38	1.13	0.88	1.04

#### Memory specificity and generalization

**Figure 3:**
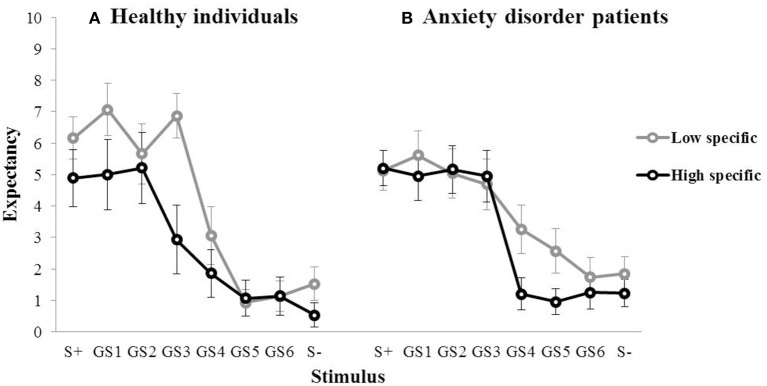
**Mean outcome expectancy ratings for S+, S−, and the six GSs (+SEM) of the first generalization test block as a function of memory specificity (high vs. low memory specificity) for (A) healthy individuals (left panel, Study 1), and (B) anxiety disorder patients (right panel, Study 2)**.

During the subsequent test of generalization, outcome expectancy ratings were obtained for the S+, the six GSs, and the S−, which are visualized for the first of three test blocks in Figure 3 (left panel). In the first generalization test block, the mean outcome expectancy ratings for the high specific participants were 4.89 (S+), 5.00 (GS1), 5.21 (GS2), 2.93 (GS3), 1.86 (GS4), 1.07 (GS5), 1.14 (GS6), 0.54 (S−). For the low specific participants, this was 6.17 (S+), 7.07 (GS1), 5.67 (GS2), 6.87 (GS3), 3.07 (GS4), 0.93 (GS5), 1.13 (GS6), 1.53 (S−). The pattern of the data, with decreases in outcome expectancy as the GSs become increasingly dissimilar to the S+, suggests the presence of generalization. The relationship between memory specificity and generalization was examined using a repeated measures MANOVA with Specificity (low specific, high specific) as between-subjects variable, and Block (Block 1–3) and Stimulus (S+, GS1, GS2, GS3, GS4, GS5, GS6, S−) as within-subjects variables. Analyses revealed a main effect of Stimulus over the three test blocks, *F*_(7,189)_ = 9.64, *p* < 0.001, partial η^2^ = 0.52, indicating the presence of generalization. There also was a main effect of Block, *F*_(2, 54)_ = 4.74, *p* = 0.018, partial η^2^ = 0.18, with an overall decrease in outcome expectancy from Block 1 to 3. However, we found no Specificity × Stimulus interaction, *F*_(7,21)_ = 1.61, *p* = 0.187, partial η^2^ = 0.06 (which again may have been due to a lack of power). In summary, we found no evidence for a relationship between memory specificity and generalization in this sample. Interestingly, we did find a main effect of Specificity over the three test blocks, *F*_(1, 27)_ = 10.49, *p* = 0.003, partial η^2^ = 0.28, with higher overall outcome expectancies in low specific individuals, relative to their high specific counterparts. Because only one S+ trial out of a total of ten trials in each test block was followed by the outcome, elevated responding to resembling stimuli may be indicative of impaired discrimination learning in low specific individuals.

### Discussion

With respect to discrimination learning, the pattern of the data suggests that high specific individuals performed better than low specific participants. However, statistical analyses provided no evidence for differences in discrimination learning as a function of memory specificity in healthy participants. The overall Specificity × Stimulus interaction was only supported at trend level, which may, however, be due to low power because of a relatively small sample size. With respect to generalization, we did not find evidence for a Specificity × Stimulus interaction. Interestingly, although we did not predict this, we found a main effect of Specificity over the three test blocks. Because only one S+ trial out of a total of 10 trials in each test block was followed by the outcome, elevated responding to resembling stimuli may be indicative of impaired discrimination learning in low specific individuals.

## Study 2

In this experiment, we tested the relationship between memory specificity and discrimination learning and generalization in a sample of individuals suffering from an anxiety disorder. In a translational framework, the demonstration of differences in associative learning as a function of memory specificity in populations suffering from psychopathology is important to establish the clinical relevance of our hypotheses. Again, we predicted that individuals low in memory specificity would show deficient discrimination learning and stronger generalization compared with high specific individuals.

### Method

#### Participants

Forty-nine individuals (30 women) diagnosed with a current DSM-IV anxiety disorder were recruited at the Academic Anxiety Centre of Maastricht (Netherlands). Diagnoses were determined by the Mini International Neuropsychiatric interview (MINI; Lecrubier et al., 1997), administered by a staff psychologist, and were reviewed in a multidisciplinary team meeting. Subsequently, all patients were assessed by a senior psychiatrist to confirm the MINI diagnosis. Table 2 gives an overview of the primary diagnoses in our patient sample. Comorbid axis 1 disorders included major depressive disorder (*n* = 26), alcohol dependence (*n* = 3), specific phobia (*n* = 3), PTSD (*n* = 2), social anxiety disorder (*n* = 2), hypochondriasis (*n* = 2), sexual dysfunction NOS (*n* = 1), and dysthymia (*n* = 1). The mean age was 41.4 (*SD* = 13.1, age range: 23–66). All participants were Caucasian and had the Dutch nationality. The study was approved by the Medical Ethics Committee, Maastricht University, The Netherlands. All participants gave informed consent.

**Table 2:** **Primary DSM-IV diagnoses for the total patient sample (Study 2)**.

	**n**	**%**
Panic disorder with agoraphobia	22	44.9
Obsessive-compulsive disorder	11	22.4
Social anxiety disorder	6	12.2
Post-traumatic stress disorder	5	10.2
Anxiety disorder due to a general medical condition	2	4.1
Specific phobia	2	4.1
Generalized anxiety disorder	1	2.0

*%, Percentage of the total patient sample (N = 49)*.

#### Apparatus and stimuli

We used the same apparatus, the same stimuli, and outcome as in Study 1.

#### Measures

In addition to the measures used in Study 1 (i.e., outcome expectancy and the AMT), we also measured trait anxiety.

#### State and trait anxiety inventory (STAI; Spielberger, 1983)

The STAI measures state (STAI-S) and trait anxiety (STAI-T) on two separate subscales, both ranging from 20 to 80, with higher scores indicating higher levels of anxiety. The Dutch version of the trait anxiety subscale by van der Ploeg (2000) was used, which has good reliability and validity.

#### Procedure

All persons suffering from an anxiety disorder were recruited at the start of their treatment, and were tested in a sound attenuated room at the Academic Anxiety Centre of Maastricht. The procedure and task sequence was identical to that of Study 1, with the associative learning task being followed by the AMT. Verbal and written instructions were provided prior to the experiment. STAI-data were collected as part of the standard intake procedure at the Academic Anxiety Centre of Maastricht.

### Results

#### Memory specificity

Because two individuals diagnosed with an anxiety disorder did not complete the AMT, analyses in this section were conducted on the remaining 47 participants. The mean proportion of specific memories was 0.58 (*SD* = 0.27, range: 0–1). Although on average, the healthy individuals reported more specific memories than the anxiety disorder patients (see Study 1), this mean difference did not reach significance, *t*_(74)_ = 1.46, *p* = 0.147. This result is consistent with previous studies, demonstrating limited memory specificity in patients with major depression and in PTSD victims, but not in patients suffering from (other) anxiety disorder (see Williams et al., 2007 for a review). However, because several patients in our sample were either diagnosed with post-traumatic stress disorder or were suffering from comorbid depression, we also investigated the evidence for limited specificity in individuals suffering from comorbid depression separately. We did not look at PTSD patients separately, as there were only five patients with PTSD as primary diagnosis. A One-Way ANOVA with group (healthy, “anxiety no-depression,” “anxiety with-depression”) as categorical predictor also yielded no evidence for differences in memory specificity between groups, *F*_(2, 73)_ = 1.18, *p* = 0.314, partial η^2^ = 0.03.

Again, a median split procedure was used to differentiate individuals high and low in autobiographical memory specificity. The mean proportion of specific memories was 0.36 (*SD* = 0.19, range: 0–0.60) for the low specific individuals (*N* = 23), and 0.79 (*SD* = 0.12, range: 0.625–1) for the high specific individuals (*N* = 24). Importantly, there was no difference in trait anxiety between the high specific (*M* = 58.9, *SD* = 9.19), and the low specific group (*M* = 55.23, *SD* = 11.12), *t*_(43)_ = 1.21, *p* = 0.231. Trait anxiety data were missing for two patients.

#### Memory specificity and discrimination learning

Visual inspection of Figure 2 (right panel) suggests a pattern in individuals suffering from an anxiety disorder similar to—if not stronger than—what was found in healthy participants, with slower (throughout acquisition) and weaker (at the end of acquisition) discrimination learning in low specific participants relative to high specific participants (means for all trials are presented in Table 1). These observations were confirmed in subsequent analyses. A repeated measures MANOVA with Specificity (low specific, high specific) as between-subjects variable, and Stimulus (S+, S−) and Trial (1–12) as within-subjects variables revealed a main effect of Stimulus, *F*_(1, 45)_ = 94.81, *p* < 0.001, partial η^2^ = 0.68, and a Stimulus × Trial interaction, *F*_(11,35)_ = 7.41, *p* < 0.001, partial η^2^ = 0.17. Importantly, we also found a Specificity × Stimulus interaction, *F*_(1,45)_ = 22.36, *p* < 0.001, partial η^2^ = 0.33, as well as a significant Specificity × Stimulus × Trial interaction, *F*_(11,35)_ = 2.24, *p* = 0.035, partial η^2^ = 0.04. In line with our hypothesis, subsequent comparisons revealed that the low specific group was slower in acquiring the S+/S− discrimination relative to the high specific group. The high specific group evidenced discrimination from the second trial onwards, *F*_(1, 45)_ = 21.94, *p* < 0.001 (*F*-values for all subsequent trials were higher). In the low specific group, however, S+/S− differentiation was only present in five out of 12 acquisition trials (i.e., 4, 6, 8, 11, 12), with significant *F*-values ranging between *F*_(1, 45)_ = 4.29, *p* = 0.044 (Trial six) and *F*_(1, 45)_ = 27.81, *p* < 0.001 (Trial 12). Moreover, whereas the low specific group clearly evidenced discrimination learning in the last two trials of acquisition, the S+/S− difference over these two trials was larger in the high specific group than in the low specific group, indicative of stronger discrimination learning in high specific patients, *F*_(1, 45)_ = 8.06, *p* = 0.007. A similar pattern emerged when analyzing outcome expectancies for the S+ and the S− separately, with lower overall responding to the S+, *F*_(1, 45)_ = 5.22, *p* = 0.027, and higher overall responding to the S−, *F*_(1, 45)_ = 12.17, *p* = 0.001, throughout acquisition in low specific relative to high specific individuals. Finally, the index of discrimination learning (see Study 1) was positively correlated with autobiographical memory specificity, *r*_(46)_ = 0.39, *p* = 0.007, indicating that individuals high in memory specificity displayed better discrimination learning throughout this phase.

#### Memory specificity and generalization

For the high specific group, the mean outcome expectancies in the first test block were 5.21 (S+), 4.96 (GS1), 5.17 (GS2), 4.96 (GS3), 1.21 (GS4), 0.96 (GS5), 1.25 (GS6), 1.23 (S−). For the low specific group, this was 5.13 (S+), 5.61 (GS1), 5.04 (GS2), 4.70 (GS3), 3.26 (GS4), 2.57 (GS5), 1.74 (GS6), 1.85 (S−; Figure 3, right panel). The same repeated measures MANOVA analyses as used in Study 1 revealed a main effect of Stimulus over the three test blocks, *F*_(7,39)_ = 14.56, *p* < 0.001, partial η^2^ = 0.50, indicating the presence of generalization. We also found a main effect of Block, *F*_(2,44)_ = 11.15, *p* < 0.001, partial η^2^ = 0.21, with an overall decrease in outcome expectancy from Block 1 to 3. Contrary to our hypothesis, however, we found no Specificity × Stimulus interaction, *F*_(7,39)_ = 1.11, *p* = 0.374, partial η^2^ = 0.02. In summary, we found no evidence for a relationship between memory specificity and generalization in this sample.

### Discussion

In a sample of patients suffering from an anxiety disorder, we found evidence for poorer discrimination learning in participants who are characterized by limited memory specificity, relative to their high specific counterparts. We found no clear evidence for differences in generalization as a function of memory specificity. The implications of these findings will be addressed in more detail in the general discussion.

Although caution is warranted when making between-study comparisons, we did not find evidence for group differences with respect to reduced memory specificity when comparing the healthy individuals from Study 1 with anxious individuals from Study 2, which is in line with previous studies. It should be noted, however, that we also did not find group differences when patients with comorbid depression were compared separately, which represents a failure to replicate a relatively robust finding in the literature (Williams et al., 2007). A possible explanation for this may be that depression was not the primary diagnosis of patients in our sample. Although speculative, autobiographical memory specificity may be different for individuals who experience a depressive episode as a consequence of or secondary to primary anxiety complaints, than for individuals for whom the primary diagnosis is MDD.

## Study 3

In addition to the relationship between memory specificity and associative learning, we were also interested in differences in associative learning between healthy individuals and individuals suffering from an anxiety disorder. As stated above, a previous investigation by Lissek and colleagues revealed impaired discrimination learning in individuals suffering from a panic disorder using a conditioning procedure with an electric shock as aversive outcome (Lissek et al., 2009). Relative to healthy participants, individuals with panic disorder reported higher outcome expectancies and showed enhanced fear potentiated startle in the presence of the S−, reflecting elevated responding to learned safety cues. In another fear conditioning experiment, Lissek et al. (2010) also demonstrated higher levels of generalization in individuals with a panic disorder, relative to healthy participants. This “overgeneralization” of conditioned fear is considered to be a pathogenic marker of panic disorder by these authors. Although fear conditioning is widely regarded as a model for the pathogenesis of anxiety disorders, it remains unclear whether these learning abnormalities in panic disorder (and other anxiety disorders, Lissek et al., 2005) are restricted to fear learning, or rather reflect a more general associative learning deficit that also manifests in fear irrelevant contexts (Meulders et al., 2013). Our associative learning task—with a non-aversive outcome—allowed us to investigate whether individuals suffering from a panic disorder or another anxiety disorder also showed impaired discrimination learning and higher levels of generalization in a neutral learning context. Therefore, we re-analyzed the data from Study 1 and Study 2 and compared performances during the learning task between the respective samples of both studies. For the sake of consistency with previous investigations, the analyses were conducted for persons with panic disorder separately, resulting in a total of three research samples for this specific research question: a “panic disorder” group (“PD group”), an “anxiety disorder—no panic” group (“no-PD anxiety group”), and a healthy community sample.

### Method

#### Participants

Of the 49 individuals suffering from an anxiety disorder, 22 (13 women) were diagnosed with panic disorder (PD group), with a mean age of 40.7 (*SD* = 12.8, age range: 23–62). Twenty-seven individuals (17 women) were diagnosed with another anxiety disorder (no-PD anxiety group). Their mean age was 42.1 (*SD* = 13.6, age range: 23–66). For a description of the diagnostic evaluation process, we refer back to Study 2. The healthy group consisted of 29 participants (22 women), with a mean age was 40.9 (*SD* = 16.9, age range: 17–86).

#### Apparatus, stimuli, measures, and procedure

As Study 3 is a re-analysis of the data collected in Study 1 and 2, we refer back to those studies for a description of the materials and the procedure.

### Results

#### Discrimination learning

**Figure 4:**
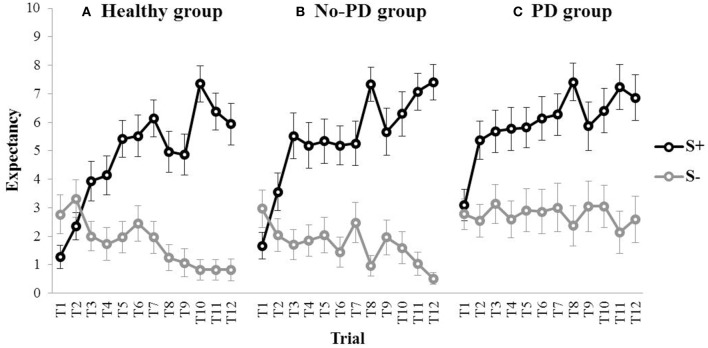
**Mean outcome expectancy ratings for S+ and S− (+SEM) throughout the discrimination learning phase (trial 1–trial 12) for (A) the healthy group, (B) the No-PD anxiety group, and (C) the PD group**.

Figure 4 shows the outcome expectancies for the S+ and the S− throughout acquisition for the healthy participants, the no-PD anxiety group, and the PD group. Visual inspection suggests discrimination learning in all three groups, with a clear increase in outcome expectancy for the S+ throughout acquisition. For the S−, however, outcome expectancies markedly decreased for the no-PD group and the healthy group, but not for the PD group. This was confirmed in statistical analyses. A repeated measures MANOVA with Group (PD group, no-PD anxiety group, healthy group) as between-subjects variable and Stimulus (S+, S−) and Trial (1–12) as within-subjects variables revealed a main effect of Stimulus, *F*_(1, 75)_ = 107.28, *p* < 0.001, partial η^2^ = 0.59, a main effect of Trial, *F*_(11,65)_ = 4.13, *p* < 0.001, partial η^2^ = 0.07, and a Stimulus × Trial interaction, *F*_(11,65)_ = 10.96, *p* < 0.001, partial η^2^ = 0.18. Interestingly, we also found a main effect of Group, *F*_(2, 75)_ = 3.79, *p* = 0.027, partial η^2^ = 0.09. Subsequent comparisons revealed that both the no-PD anxiety group, *F*_(1, 75)_ = 10.92, *p* = 0.001, and the healthy group, *F*_(1, 75)_ = 7.32, *p* = 0.008, showed a significant decrease in outcome expectancy for the S− from trial 1 to trial 12 (i.e., from 2.96 to 0.52 in the no-PD anxiety group, and from 2.76 to 0.83 in the healthy group). In the PD group, however, we found no significant decrease in outcome expectancy for the S−, *F*_(1, 75)_ = 0.05, *p* = 0.825, (i.e., 2.77 at trial 1, and 2.59 at trial 12). These results indicate that elevated responding to the S− in individuals with panic disorder is not restricted to fear conditioning, but also manifests in a neutral associative learning task.

#### Generalization

In order to investigate differences in generalization between groups, we conducted a repeated measures MANOVA with Group (PD group, no-PD anxiety group, healthy group) as between-subjects variable, and Block (Block 1–3) and Stimulus (S+, GS1, GS2, GS3, GS4, GS5, GS6, S−) as within-subjects variables. We found a main effect of Block, *F*_(2,74)_ = 12.21, *p* < 0.001, partial η^2^ = 0.17, as well as a main effect of Stimulus, *F*_(7,69)_ = 24.65, *p* < 0.001, partial η^2^ = 0.50. The analysis did not reveal a main effect of Group, *F*_(2, 75)_ = 2.13, *p* = 0.125, partial η^2^ = 0.05, or a Group × Stimulus interaction, *F*_(14,138)_ = 0.89, *p* = 0.569, partial η^2^ = 0.01. In summary, we did not find evidence for differences in generalization between patients suffering from a panic disorder or another anxiety disorder, and healthy participants.

### Discussion

Re-analysis of the data from Study 1 and Study 2 showed that individuals with panic disorder reported higher outcome expectancies for the S− relative to healthy participants and individuals suffering from another anxiety disorder. In fact, their outcome expectancy for the S− did not decrease and remained at the same level throughout acquisition. These data replicate previous findings by Lissek et al. (2009), who also demonstrated elevated responding to the S− in patients with panic disorder. However, because this study used a neutral outcome instead of an aversive US, these results also put previous findings in a different perspective. Whereas, Lissek and colleagues termed the higher levels of responding to the S− as impaired safety learning, danger vs. safety learning does not apply to a neutral associative learning task. These data suggest that learning abnormalities found in panic disorder may not be restricted to fear learning, but rather reflect a more general associative learning deficit that also manifests in fear irrelevant contexts. More specifically, individuals with panic disorder appear to overestimate the probability of an outcome in situations (e.g., after S− presentations) other than the situation in which the outcome was originally experienced (e.g., after S+ presentations). To the extent that these situations share stimulus properties, as was the case in this study, we could also regard this as a form of overgeneralization, but not restricted to fear. Future studies should aim to assess the breadth of learning anomalies in panic disorder and other anxiety disorders. A limitation of this study was that participants in the healthy group were not subjected to the full MINI-interview, but were only interviewed about any past or current anxiety or depressive disorder. However unlikely, the conclusions of this study may be compromised if this group included patients suffering from panic disorder who did not report this (i.e., false negative inclusion in the healthy sample), and who did not show a learning deficit similar to what was found in the sample of patients suffering from a panic disorder. Further, it is noteworthy that female participants were somewhat overrepresented in the healthy group, relative to the PD group and the no-PD anxiety group. To our knowledge, however, there is no evidence for sex differences in neutral associative learning procedures. Future studies should assess whether these learning abnormalities also manifest in neutral learning contexts in other anxiety disorders. In this study, the sample size of the no-PD anxiety group did not allow us to investigate other anxiety disorders separately, but it is plausible that differences in (neutral) associative learning between other clinical groups within the heterogeneous category of anxiety disorders exist.

## General discussion

In the present paper, we investigated the relationship between autobiographical memory specificity and discrimination learning and generalization in an associative learning task. Limited memory specificity was associated with poorer discrimination learning in a sample of patients suffering from an anxiety disorder. In a sample of healthy participants, a similar pattern was found but was not significant (possibly due to a lack of power). We found no clear evidence for differences in generalization as a function of memory specificity. Further, results showed that individuals suffering from a panic disorder displayed elevated responding to the S− in a neutral associative learning task, relative to healthy participants and individuals suffering from other anxiety disorders.

This series of studies demonstrates that autobiographical memory specificity is related to individual differences in novel learning in patients suffering from an anxiety disorder. Although all participants were exposed to the same learning experience, participants high in memory specificity displayed faster and stronger discrimination learning relative to their low specific counterparts. Accurate discrimination learning requires the encoding into memory and the retrieval from memory of information that is sufficiently specific to allow differentiation between similar past experiences. In this study, accurate discrimination was not only determined by perceptually differentiating the S+ from the S−, but also by correctly identifying the associative relationships between the S+, the S−, and the outcome. It is possible that participants low in memory specificity experienced more difficulty in encoding and/or retrieving this event-specific information from memory throughout acquisition. These results indicate that impaired discrimination learning, which has been shown in patients suffering from an anxiety disorder, may be—in part—due to limited autobiographical memory specificity. In a limited number of studies, individuals suffering from clinical or subclinical anxiety evidenced no impairments in discrimination learning (Indovina et al., 2011; Torrents-Rodas et al., 2013). Our data suggest that abnormalities in discrimination learning may only be found in a subset of individuals who are characterized by limited memory specificity.

It is important to note, however, that these data do not allow causal inferences. Low memory specificity may impair discrimination learning, but the reverse may also be true. To the extent that learning experiences are not accurately differentiated from one another, they will also not be encoded as unique, specific events into memory. Further, it is possible that a third variable affects both autobiographical memory specificity and discrimination learning. For instance, previous studies have shown that performance on the AMT is associated with measures of executive control such as verbal fluency (Dalgleish et al., 2007). Impaired executive control may account for both reduced memory specificity and deficits in discrimination learning. If this would be the case, it still applies that cognitive variables should be taken into account in the study of associative learning phenomena. Future investigations should aim to unravel the causal relationships between memory specificity and discrimination learning, for instance by experimentally manipulating memory specificity or working memory.

With respect to generalization, a previous investigation conducted in our lab (Lenaert et al., 2012) revealed that first year psychology students who were low in memory specificity generalized more than students high in memory specificity. In the present paper, this effect could not be replicated. Besides a possible lack of power, there also were methodological differences between studies. Because student populations are known to respond highly specifically to the standard AMT, a minimal instructions version of the AMT was used in that study (Debeer et al., 2009). In that particular version, the instruction to come up with specific memories, as well as the provision of examples of specific and non-specific memories are omitted. This way, inter-individual variability in memory specificity is enhanced. As a consequence, however, these studies are not readily comparable. It is possible that group differences in generalization as a function of memory specificity are only observed when assessing the “default mode” of memory recall, as is done by the minimal instructions AMT. On the other hand, if participants are explicitly instructed to come up with a specific memory like in the present series of studies, otherwise low specific individuals may now generate more specific memories as well, resulting in less inter-individual variability in memory specificity to explain differences in generalization. Of course, we must also take into account the possibility that this failure to replicate previous findings can be due to random sampling from a population where the true effect size of memory specificity on generalization is rather small (Francis, 2012). A limitation of both Study 1 and 2 is that we used a median split procedure to differentiate individuals high and low in memory specificity, in that this procedure omits a sizable amount of the variance present in the data. With respect to discrimination learning, we therefore used an additional approach which allowed us to investigate the relation between memory specificity and discrimination learning in a continuous fashion.

In summary, we demonstrated that autobiographical memory specificity was related to discrimination learning in an associative learning task in a sample of anxiety disorder patients. In a healthy community sample, a similar but not significant pattern emerged. These results emphasize the importance of incorporating cognitive variables in associative learning theories and their implications for the development of anxiety disorders. Based on individual differences in cognitive variables that may influence learning, similar experiences may lead to different outcomes. This study represents a first stepping stone for future investigations of this relationship by experimentally manipulating memory specificity, by using other learning procedures, and by assessing other dependent variables such as fear potentiated startle and avoidance behavior. Further, the finding that learning abnormalities in patients suffering from a panic disorder also manifest in fear irrelevant contexts puts previous findings in a different perspective, and should stimulate future research efforts into the breadth of associative learning deficits in panic disorder and other anxiety disorders.

## Funding

This research was supported by the Center for Excellence on Generalization Research (GRIP × TT; University of Leuven grant PF/10/005). BL is a research assistant for the FWO-Flanders.

## Author contributions

BL, DH, and KS developed the study concept, and contributed to the study design. Testing and data collection were performed by BL. BL performed the data analysis and interpretation under the supervision of DH, KS, and BV. BL drafted the paper, and YB, KS, DH, and BV provided critical revisions. All authors approved the final version of the paper for submission.

## Conflict of interest statement

The authors declare that the research was conducted in the absence of any commercial or financial relationships that could be construed as a potential conflict of interest.

## Footnotes

^1^The Dutch words were respectively: aangenaam, boos, belangstellend, gekwetst, trots, kwaad, sociaal, onhandig, enthousiast, ontgoocheld.
